# LGR6 is a prognostic biomarker for less differentiated tumors in lymph nodes of colon cancer patients

**DOI:** 10.3389/fonc.2024.1393075

**Published:** 2024-04-23

**Authors:** Hagar Eltorky, Manar AbdelMageed, Hager Ismail, Faten Zahran, Adel Guirgis, Lina Olsson, Gudrun Lindmark, Marie-Louise Hammarström, Sten Hammarström, Basel Sitohy

**Affiliations:** ^1^ Department of Clinical Microbiology, Umeå University, Umeå, Sweden; ^2^ Department of Diagnostics and Intervention, Umeå University, Umeå, Sweden; ^3^ Department of Biochemistry, Faculty of Science, Zagazig University, Zagazig, Egypt; ^4^ Department of Pathology, Faculty of Veterinary Medicine, Zagazig University, Zagazig, Egypt; ^5^ Department of Clinical Pathology, Faculty of Veterinary Medicine, Zagazig University, Zagazig, Egypt; ^6^ Department of Molecular Biology, Genetic Engineering, and Biotechnology Research Institute, University of Sadat City, Sadat, Menoufia, Egypt; ^7^ Institution of Clinical Sciences, Lund University, Lund, Sweden

**Keywords:** colon cancer, regional lymph nodes, cancer stem cells, LGR6, LGR5, CEA, CXCL16, qRT-PCR

## Abstract

**Introduction:**

The aim was to investigate whether the stem cell marker LGR6 has prognostic value in colon cancer, alone or in combination with the prognostic biomarkers CEA and CXCL16.

**Methods:**

LGR6 mRNA levels were determined in 370 half lymph nodes of 121 colon cancer patients. Ability to predict relapse after curative surgery was estimated by Kaplan-Meier survival model and Cox regression analyses.

**Results:**

Patients with high LGR6 levels [LGR6(+)] had a decreased mean survival time of 11 months at 5-year follow-up and 47 months at 12-year follow-up, respectively, with hazard ratios of 3.2 and 2.8. LGR6 mRNA analysis added prognostic value to CEA and CXCL16 mRNA analysis. In the poor prognosis groups CEA(+) and CXCL16(+), further division was achieved by LGR6 analysis. LGR6(+) patients had a very poor prognosis. LGR6 also identified a small number of CEA(-), TNM stage I patients who relapsed suggesting stem cell origin of these tumors. LGR6 and LGR5 levels correlated strongly in lymph nodes of stage I and IV patients but not in stage II patients, suggesting that these stem cell markers are differentially regulated.

**Conclusion:**

This study highlights LGR6 as a useful prognostic biomarker independently and in combination with CEA, CXCL16 or LGR5 identifying different risk groups.

## 1 Introduction

Colorectal cancer (CRC) is a leading cause of cancer-related deaths worldwide and a form of cancer that is increasing in frequency (1). The main treatment modality for CRC is surgery with its risk of postoperative complications of which surgical site infection (SSI) is the most common (2, 3). Unfortunately, approximately 25% of patients having curative surgery will relapse and most of them will die from cancer (4, 5). Since the standard methods are not able to identify this group of patients there is an urgent need to develop methods that can accomplish this aim. The standard method to determine if the tumor has spread to the regional lymphatic field is histopathology. Although still considered the most important method to identify patients with tumors that will relapse, the method is far from perfect. Thus, a significant fraction of patients judged to be free of tumors in their lymph nodes (TNM stage I and II patients) actually contain tumor cells that are missed by histopathology. The main reasons for this are that only a small fraction of the lymph node (LN) volume is analyzed, and that histopathology is a subjective method requiring a trained pathologist. Biomarker mRNA analysis is a very promising alternative allowing analysis of the entire LN volume and analysis of a combination of biomarkers that characterize different properties of the tumors that can be combined in a kit. ColoNode, which combines analysis of mRNAs of CEA (CEACAM5), Kallikrein Related Peptidase 6 (KLK6), Solute Carrier Family 35 Member D3 (SLC35D3), Mucin 2 (MUC2) and Periostin (POSTN) of half the LN volume is a successful colon cancer (CC) prognostic test that surpasses histopathology in identifying patients that will relapse and in addition grades patients with different degrees of risk (6). The study identified two distinct group of patients, one which should be recommended postoperative adjuvant treatment and another which should be left untreated. There is, however, a small group who are tumor cell positive in the LNs but the tumor cells do not demonstrate all aggressiveness factors. For this group no clear treatment recommendation can be given. Analysis of markers for cancer stem cells (CSC) may help in dividing this group further.

CRC may originate from epithelial stem cells or from more mature epithelial cells, and tumors in a patient may be a mixture of tumor cells of both origins. Moreover, different patients are likely to differ in the proportion of stem cell derived tumors. CSC are considered to be more aggressive than other cancer cells and have self-renewal and multi-lineage differentiation capacities and play important roles in tumor initiation, progression, metastasis, drug and radiation resistance (7–9). CSC can be identified by biomarkers. We have recently studied the CSC biomarkers leucine-rich repeat-containing G protein-coupled receptor 4 and 5 (LGR4 and LGR5) in CC and found both markers to be associated with poor prognosis after curative surgery when applied to LN mRNA analysis (10). Additionally, we found that the chemokine CXCL17 and the G protein-coupled receptor 35 (GPR35) were associated with stem cell-like features, detecting undifferentiated CC tumor cells (11–13).

The LGR subfamily contains three members, LGR4, LGR5 and LGR6. They are members of the glycoprotein hormone receptor subfamily of rhodopsin-like, seven transmembrane domain receptors (14). All three LGRs function as receptors for the R-spondin family of stem cell factors to potentiate Wnt/β-catenin signaling (15–18). The R-spondins (RSPO1-4) are secreted proteins. For example, LGR6 is a high affinity receptor for RSPO1-3 and binding has a positive effect on Wnt/β-catenin signaling (18). Not only do the LGRs interact with RSPOs but also with each other - interaction score between LGR6 and LGR5 or LGR4 respectively >0.905 (= very high confidence) (19). A recent study showed that LGR6 also activates the PI3K/AKT pathway in CRC (20). Several groups have studied the prognostic value of LGR6 in cancer. In esophageal squamous cell carcinoma patients high levels of LGR6 in the primary tumor indicated significantly worse prognosis than patients with low levels (19). In CRC one study gave the same result, that is, that patients with high levels of LGR6 have significantly shorter overall survival rates than patients with low levels (20), while another study showed the opposite result (21). Targeting CSC in CRC may constitute a new and effective treatment strategy (22).

Here, we have studied the prognostic value of the CSC marker LGR6 for analysis of regional LNs of CC patients. Analyses have been performed at the mRNA level using a novel highly specific quantitative reverse transcriptase polymerase chain reaction (qRT-PCR) assay for LGR6, that detects all 3 splice forms of LGR6 mRNA. The same clinical material of LNs from CC patients as has been investigated earlier for expression of CEA-, LGR4-, LGR5- and the chemokine CXCL16- mRNAs has been used (10, 23). The utility of combining expression levels of the different biomarkers was also investigated. We found that high mRNA levels in lymph nodes of LGR6 predict shortened disease-free survival and that determinations of LGR6 together with the CC prognostic markers CEA and CXCL16 significantly enhances prognostic effectiveness.

## 2 Materials and methods

### 2.1 Patients and tissue specimens for mRNA analysis

Primary tumor specimens were gathered from 66 CC patients (30 men and 36 women; median age 74 years, range 42–88 years). Patients belonged TNM stages as follows: 14 patients in stage I, 30 patients in stage II, 17 patients in stage III, and 5 patients in stage IV. None of the patients received preoperative therapy. The specimens were collected immediately after resection, frozen and preserved at -70°C until RNA extraction. Normal colon specimens were taken from the resection margins of tumors of 30 CC patients (17 men and 13 women; median age 72 years, range 57–85 years).

Half LNs were gathered from 121 CC patients (55 men and 66 women; median age 74 years, range 42–89 years). Of these, 69 LNs came from 23 patients in stage I, 186 LNs from 52 patients in stage II, 85 LNs from 37 patients in stage III, and 30 LNs from 9 patients in stage IV. According to routine histopathology, disseminated tumor cells were detected in 20 LNs [H&E(+)] and 350 LNs were H&E(-). Thirteen non-cancer patients (10 males and 3 women; median age 23 years, range 9–32 years) provided 77 control LNs. One control patient had lipoma, 1 had Crohn’s disease, and 11 had ulcerative colitis. The half LNs were collected immediately after resection, frozen and preserved at -70°C until RNA extraction.

### 2.2 Cell lines

RNA from 5 human CC cell lines (HT29, LS174T, Caco2, T84, HCT8), 1 T cell line (Jurkat), 2 B cell lines (CNB6, KR4), 1 monocyte cell line (U937), 1 endothelial cell line (HUVEC) and primary foreskin fibroblasts (FSU) were from previous studies (24–29).

### 2.3 Real-time qRT-PCR

For absolute quantification of LGR6 mRNA, we constructed a real time qRT-PCR assay using specific primers placed in different exons and a reporter dye-labeled probe hybridizing over the exon boundary in the amplicon and specific RNA copy standard. The LGR6 mRNA assay detects all three known transcript variants (NM_001017403.2, NM_021636.3, NM_001017404.2). The primer and probe sequences were: forward primer 5′-AGCTGGAGATGGAGGACTCAAA-3′, reverse primer 5′-CCAGCTTTCAAAGAGGTACTCACA-3′, and probe 5′-TACTCCAGGCCCCTTC-3′. MGB served as the quencher dye and FAM as the reporter dye. The amplicon measured 95 bases. The qRT-PCR profile was 60°C for 5 min and 95°C for 1 min, followed by 45 cycles of 95°C for 15 s and 60°C for 1 min. The RNA copy standard was a custom synthesized RNA oligonucleotide (Dharmacon, Lafayette, CO, USA) with identical sequence to the area amplified in the qRT-PCR assay. Real-time qRT-PCR assays for CEA, CXCL16, LGR4 and LGR5 mRNAs were described previously (10, 23, 24). Each qRT-PCR run included serial dilutions of the respective RNA copy standard at concentrations ranging from 10^3^ to 10^8^ copies/µL. Concentrations in unknown samples were determined from the standard curve and expressed as copies of mRNA/µL. The concentration of 18S rRNA was expressed as arbitrary units from a standard curve of serial dilutions of a preparation of total RNA from human peripheral blood mononuclear cells. One unit was defined as the amount of 18S rRNA in 10 pg RNA (30). Expression levels were expressed as mRNA copies/18S rRNA unit.

### 2.4 Statistical analysis

The statistical significance of differences between LGR6 mRNA levels in primary CC tumors compared to normal colon tissues, H&E(+) LNs compared to H&E (-) LNs, LNs of patients in different TNM stage groups, and LNs in the CEA(+), CEA(int) and CEA(-) groups were analyzed using two-tailed Mann-Whitney rank sum test. Correlations between LGR6 mRNA levels and CEA, CXCL16, LGR4 and LGR5 mRNA levels were analyzed using the nonparametric Spearman correlation coefficient. The software utilized for statistical calculations was GraphPad Prism 9 (Graph pad Software, San Diego, CA, USA). The SPSS software (IBM Corporation, Armonk, NY, USA) was used for statistical analyses of differences between patient groups in disease-free survival time and analyses of risk for recurrent disease after surgery, according to the Kaplan-Meier survival model in combination with the log-rank test and univariate Cox regression analysis. A *p*-value of ≤ 0.05 was considered statistically significant.

### 2.5 Ethical considerations

All procedures performed in studies involving human participants were in accordance with the ethical standards of the institutional research committee and with the 1964 Helsinki Declaration and its later amendments and comparable ethical standards. Tumor samples and LNs were collected after patients’ written, informed consent. The study was approved by the Local Ethics Research Committee of the Medical Faculty, Umeå University, Umeå, Sweden (registration number: 03-503; date of approval: 3 December 2003 and registration number: 2023-01396-01; date of approval; 3^rd^ of May 2023).

## 3 Results

### 3.1 Expression levels of LGR6 mRNA in primary colon cancer tumor, normal colon tissue, colon cancer cell lines and immune cell lines

**Figure 1:**
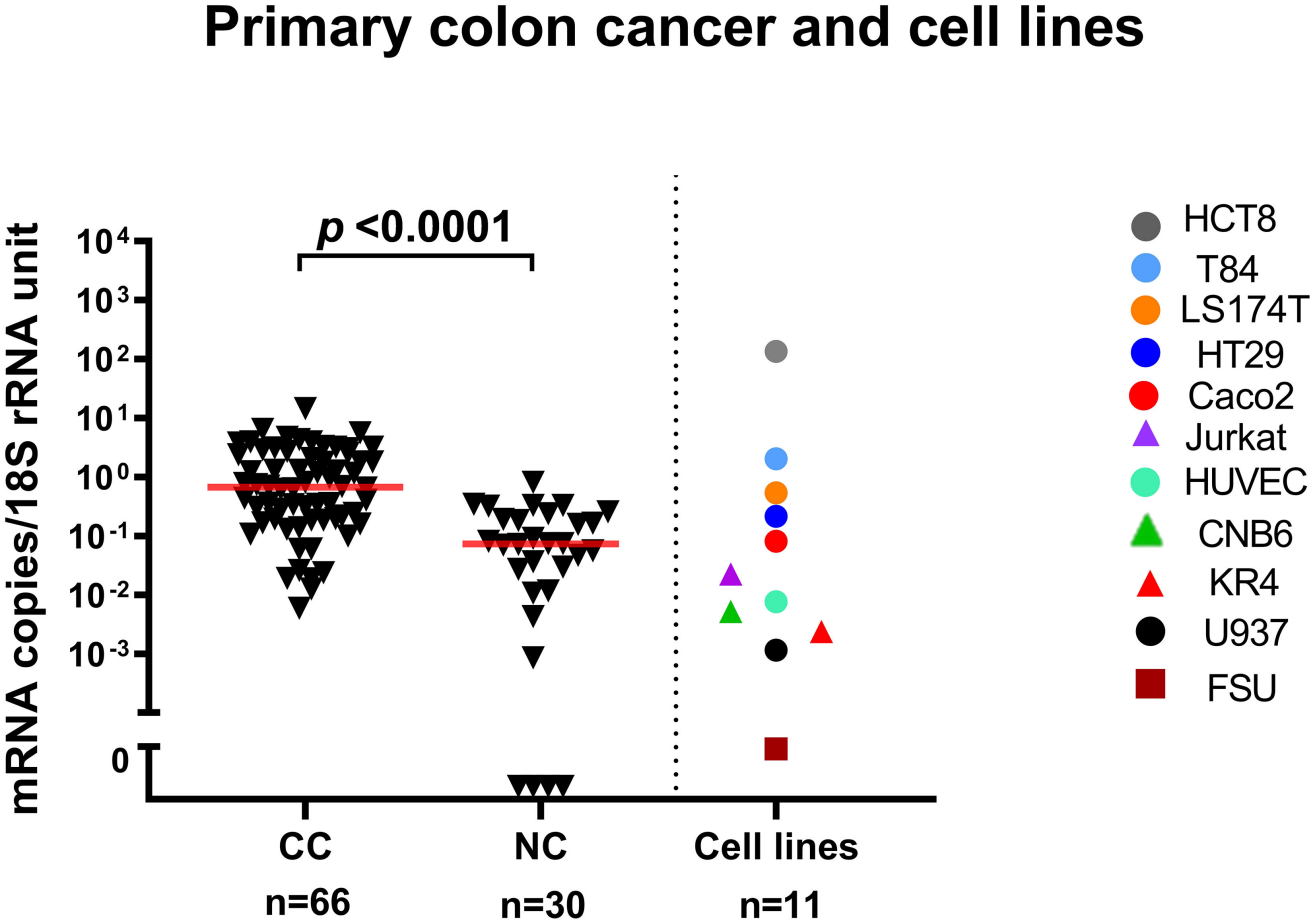
LGR6 mRNA expression levels in primary colon cancer tissues (CC), resected normal colon tissues (NC), and in a panel of colon cancer cell lines (HCT8, T84, LS174T, HT29, Caco2), a T-cell line (Jurkat), an endothelial cell line (HUVEC), two B-cell lines (CNB6 and KR4), a monocyte cell line (U937), and primary foreskin fibroblast cells (FSU). The median values are indicated by the red horizontal lines. The n-values show the number of analyzed primary CC tumors, normal colon samples and cell lines. Two-tailed Mann-Whitney rank sum test was used to determine the *p*-value.

The LGR6 mRNA median expression level was ten times higher in primary tumor (CC) than in normal colon tissues (NC) (0.7 and 0.07 mRNA copies/18S rRNA unit, respectively; *p* < 0.0001. **Figure 1**). The expression levels in four of five CC cell lines (T84, LS174T, HT29, CaCo2) were similar to those of primary CC tumors. In the fifth CC cell line (HCT8) the level was almost 100 times higher. Immune cell lines expressed clearly lower levels of LGR6 mRNA than CC cell lines of which the T cell line, Jurkat, expressed the highest level (about 0.03 mRNA copies/18S rRNA unit). Only very low levels of LGR6 mRNA were expressed in an endothelial cell line (HUVEC) and no LGR6 mRNA was detected in foreskin fibroblasts (FSU) (**Figure 1**). In a LN context, LGR6 mRNA can therefore be classified as epithelial cell specific with minimal influence of other cells that occurs in this organ.

### 3.2 Expression levels of LGR6 mRNA in regional lymph nodes of colon cancer patients

**Figure 2:**
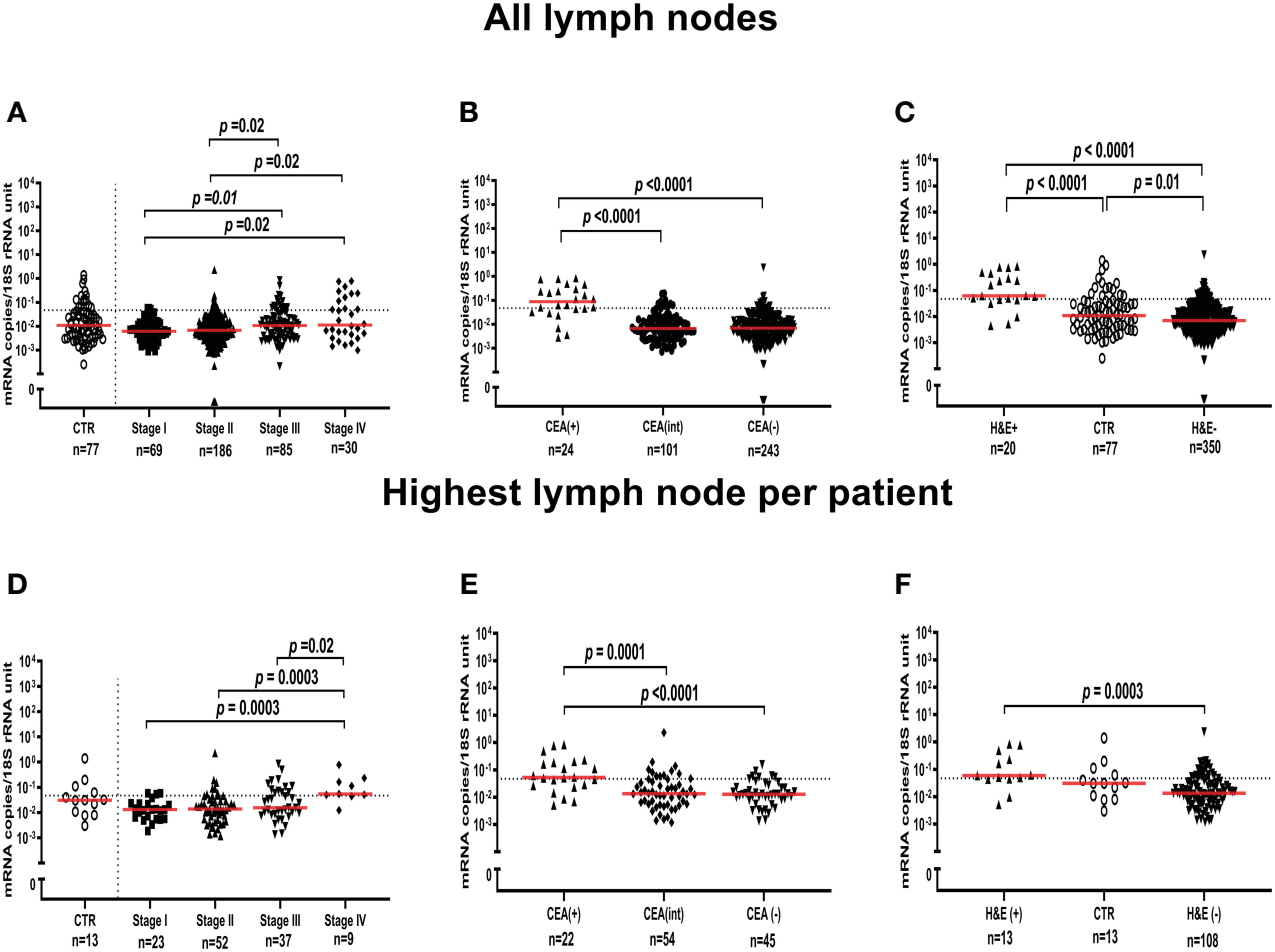
LGR6 mRNA expression levels in all lymph nodes **(A-C)** and the lymph node with the highest level for each patient **(D-F)**. **(A, D)** show LGR6 levels in lymph nodes from non-cancerous disease patients (CTR) and from colon cancer patients of different TNM stages (Stage I-IV). **(B, E)** show LGR6 levels in lymph nodes grouped according to their level of CEA mRNA, CEA(+) (> 3.67 copies/18S rRNA unit), CEA(int) (between 0.013 and 3.67 copies/18S rRNA unit) and CEA(-) (<0.013 copies/18S rRNA unit). **(C, F)** shows LGR6 levels in metastatic lymph nodes [H&E(+)], non-metastatic lymph nodes [H&E(-)] and non-cancerous disease patients (CTR). Dashed horizontal line indicates the 75^th^ percentile (0.0471 LGR6 mRNA copies/18S rRNA unit) which was used as cut-off for LGR6(+) and LGR6(-) categories in analyses of prognostic value. The n-values shown the number of lymph node samples analyzed. Two-tailed Mann-Whitney rank sum test was used to determine the *p*-values.

The mRNA expression levels of LGR6 were evaluated in a panel of 370 regional LNs from 121 CC patients representing all four TNM-stages and 77 LNs from 13 patients with non-cancerous disease. LGR6 mRNA median expression levels were 0.006, 0.007, 0.011 and 0.011 mRNA copies/18S rRNA unit in TNM stages I, II, III and IV, respectively. Notably, the LGR6 mRNA median expression level was 0.011 mRNA copies/18S rRNA unit in LNs of control patients. There was a significant difference of LGR6 mRNA expression level between LNs of stage I and stage III (*p*=0.01), between stage I and stage IV (*p*=0.02), between stage II and stage III (*p*=0.02), and a significant difference between LNs of stage II and stage IV (*p*=0.02) (**Figure 2A**).

LGR6 mRNA expression levels were then compared with CEA mRNA levels previously determined for the same 370 LNs (24). **Figure 2B** shows the result. LNs were divided into 3 groups depending on the CEA mRNA levels. CEA(+) >3.67 copies/18S rRNA unit, CEA(int) <3.67 and >0.013 copies/18S rRNA unit, and CEA(-) <0.013 copies/18S rRNA unit. The LGR6 mRNA median expression levels were 0.09, 0.007 and 0.007 mRNA copies/18S rRNA unit in the CEA(+), CEA(int) and CEA(-) LN groups, respectively. A highly significant difference between the expression levels of the CEA(+) and both the CEA(int) and CEA(-) groups was seen (*p*<0.0001).

In **Figure 2C** the LGR6 mRNA expression levels were compared with the results of examination for presence of tumor cells of H&E stained LN tissue sections of the same 370 LNs of CC patients and in 77 LNs of controls. Twenty LNs had metastases [H&E(+)] and 350 LNs were H&E(-). The LGR6 mRNA median expression level was 9 times higher in H&E(+) than H&E(-) LNs (0.06 and 0.007 mRNA copies/18S rRNA unit, respectively). There was a highly significant difference between H&E(+) LNs and both H&E(-) LNs and LNs of control patients (*p*<0.0001 and p<0.0001, respectively).

In order to make the LGR6 mRNA expression data directly comparable with the survival data as determined by Cox regression and Kaplan-Meier analysis (see below) we used the LGR6 mRNA level of the LN expressing the highest level to represent each patient. **Figures 2D-F** show the results for the 121 CC patients and the 13 control patients. As can be seen the expression pattern was closely similar to that found when all LNs were analyzed. One difference was, however, that control LNs did not differ significantly from H&E(+) or H&E(-) LNs, although the trend was the same, that is, to express levels in-between the other two groups (**Figure 2F**).

### 3.3 Correlation between mRNA expression levels of LGR6 and of LGR4, LGR5, CEA and CXCL16 in regional lymph nodes of colon cancer patients

The mRNA expression levels of LGR4, LGR5, CEA and CXCL16 have previously been determined in the same 370 LNs studied in this work (10, 23, 24). **Table 1** shows the correlation coefficients (r) and the degree of significance of the correlation between the biomarker mRNAs for all 121 CC patients both for the patients as one group and for the different TNM stage groups. The highest correlation coefficient was seen between LGR6 and CEA (r=0.73), followed by LGR6 and CXCL16 (r=0.66), and thereafter LGR6 and LGR5 (r=0.53) in LNs of stage IV patients. All three correlations were highly significant. Significant correlation between LGR6 and CEA and between LGR6 and CXCL16 was also seen in LNs of stage III patients. These data indicate that LGR6, CEA, CXCL16 and LGR5 to a large extent identifies the same tumor cell population which is enriched in LNs of stage III and IV patients.

**Table 1:** Correlations between LGR6 mRNA expression levels and expression levels of LGR4, LGR5, CEA and CXCL16 mRNAs in lymph nodes of colon cancer patients.

			LGR4	LGR5	CEA	CXCL16
LGR6	**All CC LNs**	**r**	0.28	0.22	0.12	0.33
		***p*-value**	<0.0001	<0.0001	0.02	<0.0001
	**TNM Stage I LNs**	**r**	0.37	0.37	-0.09	0.34
		***p*-value**	0.002	0.002	0.49	0.004
	**TNM Stage II LNs**	**r**	0.23	0.04	-0.11	0.26
		***p*-value**	0.002	0.55	0.15	0.0003
	**TNM Stage III LNs**	**r**	0.19	0.26	0.35	0.34
		***p*-value**	0.08	0.02	0.001	0.002
	**TNM Stage IV LNs**	**r**	0.40	0.53	0.73	0.66
		***p*-value**	0.03	0.003	<0.0001	<0.0001

The correlation coefficients (r) and the *p*-values were calculated by two-tailed Spearman’s rank order correlation test.

### 3.4 Clinical utility of expression level analysis of LGR6 mRNA alone or in combination with CEA or CXCL16 mRNA in lymph nodes to predict colon cancer recurrence after surgery

**Figure 3:**
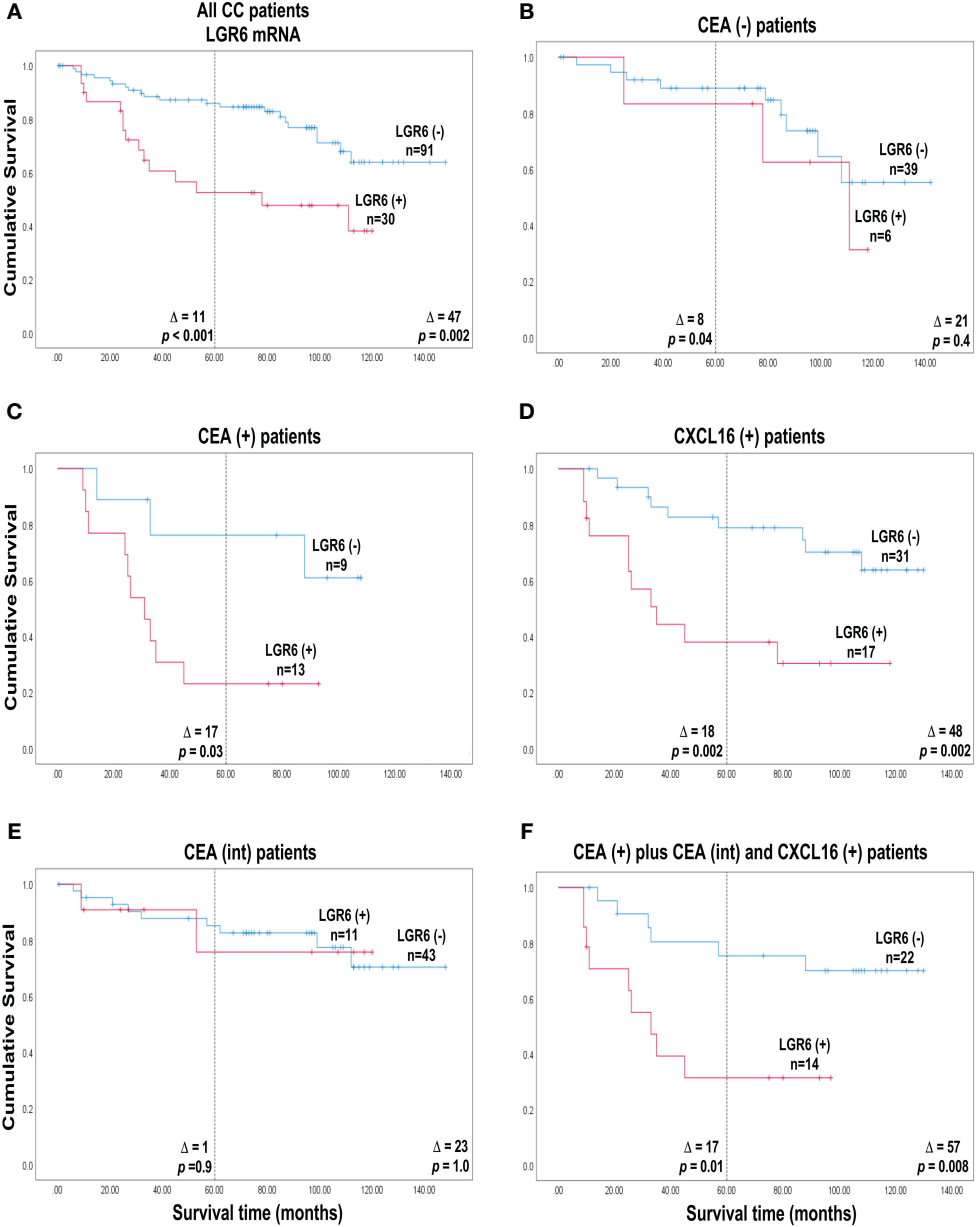
Cumulative survival curves according to Kaplan-Meier of colon cancer patients belonging to either of the two categories LGR6(+) and LGR6(-) defined as patients with a LGR6 mRNA level in the highest lymph node above respectively below the cut-off 0.0471 LGR6 mRNA copies/18S rRNA unit. **(A)** all 121 colon cancer patients. **(B)** the 45 CEA(-) patients who had CEA mRNA levels <0.013 copies/18S rRNA unit. **(C)** the 22 CEA(+) patients who had CEA mRNA levels >3.67 copies/18S rRNA unit. **(D)** the 48 CXCL16(+) patients who had CXCL16 mRNA levels >7.2 copies/18S rRNA unit. **(E)** the 54 CEA(int) patients who had CEA mRNA levels between 0.013 and 3.67 copies/18S rRNA unit. **(F)** the 36 patients who had CEA mRNA levels above 0.013 copies/ 18S rRNA unit and CXCL16 levels >7.2 copies/18S rRNA unit. The numbers next to the curves indicate the number of patients in the category. The difference between mean survival time without recurrence between the categories are given as Δ-values. The *p*-values are from log rank test analysis of survival data. The dashed line indicates 5 years of observation after surgery.

To evaluate the significance of using the expression levels of LGR6 mRNA in regional LNs of CC patients for prediction of disease recurrence after surgery, we used Cox regression analysis to calculate the hazard risk ratio for recurrence and Kaplan-Meier survival model combined with the log-rank test to evaluate differences in disease-free survival time after surgery. Each patient was represented by the LN with the highest expression level of LGR6 mRNA. A cut-off level discriminating between patients with high and low risk for recurrence was analytically determined, dividing the patients into two categories, LGR6(+) and LGR6(−). The cutoff used to divide the patients into a LGR6(+) category and a LGR6(-) category was the 75^th^ percentile (0.0471 LGR6 mRNA copies/18S rRNA unit). The prognostic value of the LGR6 level in the CEA(+)-, CEA(int)-, CEA(-)-, and CXCL16(+) groups, as well as in a of group of patients that were CEA(+)-,CEA(int)- and CXCL16(+) was also investigated. These survival analyses are shown in **Table 2** and Kaplan–Meier cumulative survival curves in **Figure 3**.

**Table 2:** Comparative analysis of average survival time after surgery and risk of recurrence of disease in colon cancer patients with LGR6(+) or LGR6(-) lymph nodes.

Patient Group	Category^a^	Number of patients	5-Year Follow Up after Surgery					12-Year Follow Up after Surgery				
			Disease-free survival time^b^			Risk of recurrence of CC^c^		Disease-free survival time^b^			Risk of recurrence of CC^c^	
			Average (months)	Difference (months)	*p*-value	Hazard Ratio (95% CI)	*p*- value	Average (months)	Difference (months)	*p*-value	Hazard Ratio (95% CI)	*p*- value
All CC Patients	LGR6(-)	91	54	11	<0.001	3.2 (1.6-6.4)	0.001	119	47	0.002	2.8 (1.1-5.4)	0.003
	LGR6(+)	30	43					72				
CEA(+) patients^d^	LGR6(-)	9	49	17	0.03	3.7 (1.0-13.6)	0.05					
	LGR6(+)	13	32									
CEA(int) patients^e^	LGR6(-)	43	54	1	0.9	0.9 (0.2-4.1)	0.9	122	23	1.0	1.0 (0.2-4.7)	1.0
	LGR6(+)	11	53					99				
CEA(-) patients^f^	LGR6(-)	39	55	8	0.04	3.8 (1.0-15.4)	0.05	112	21	0.4	1.7 (0.5-6.3)	0.4
	LGR6(+)	6	47					91				
CXCL16(+) patients^g^	LGR6(-)	31	53	18	0.002	3.9 (1.5-9.7)	0.004	104	48	0.002	3.7 (1.5-9.2)	0.004
	LGR6(+)	17	35					56				
CEA(int)/CEA(+)/ CXCL16(+) patients^h^	LGR6(-)	22	52	17	0.01	3.5 (1.2-9.8)	0.02	103	57	0.008	3.8 (1.3-10.7)	0.01
	LGR6(+)	14	35					46				

a CC patients were divided into categories according to LGR6 mRNA level. LGR6(-): the highest lymph node had <0.0471 mRNA copies/18S rRNA unit; LGR6(+): the highest lymph node had ≥0.0471 mRNA copies/18S rRNA unit.

b Mean survival time after surgery calculated by cumulative survival analysis according to the Kaplan-Meier model.

c Hazard ratio with 95% confidence interval (CI) calculated according to univariate Cox regression analysis.

d CC patients with CEA mRNA levels above 3.67 mRNA copies /18S rRNA unit.

e CC patients with CEA mRNA levels between 0.013 and 3.67 mRNA copies /18S rRNA unit.

f CC patients with CEA mRNA levels below 0.013 mRNA copies /18S rRNA unit.

g CC patients with CXCL16 mRNA levels above 7.2 mRNA copies /18S rRNA unit.

h CC patients with CEA mRNA levels above 0.013 mRNA copies /18S rRNA unit and CXCL16 mRNA levels above 7.2 mRNA copies /18S rRNA unit.

Patients in the LGR6(+) category (*n* = 30) showed a 3.2-fold higher risk of recurrence compared to the LGR6(-) category (*n* = 91) when followed for five years and a 2.8-fold higher risk at a follow-up time of 12 years (*p*=0.001 and *p*=0.003, respectively). According to Kaplan-Meier survival analysis the associated decrease in mean disease-free survival time was 11 months at 5 years and 47 months at 12 years after surgery (*p*<0.001 and *p*=0.002, respectively, **Figure 3A**).

A clear-cut division of the patients in terms of survival was seen if LGR6 mRNA analysis was combined with CEA mRNA analysis. Thus, when patients in the CEA(+) group were divided into a LGR6(+) category (*n* = 13) and a LGR6(-) category (*n* = 9) a markedly increased risk for recurrence with a hazard ratio (HR) of 3.7 was seen for the positive category when followed for five years (*p*=0.05). Note, that no patients were alive in the LGR6(+) group 90 months after surgery. Note also that patient survival in the LGR6(-) category was poor although not as poor as in the LGR6(+) category of CEA(+) patients. The associated decrease in mean survival time was 17 months in 5 years (*p*=0.03; **Figure 3C**). In contrast, no significant difference in recurrence risk or mean survival time between the LGR6(+) and LGR6(-) categories was observed when analysis was confined to LNs of the CEA(int) group (**Figure 3E**). For the CEA(-) group there was a small but significant difference with the LGR6(+) category having a worse outcome than the LGR6(-) category at 5-years follow-up. (**Figure 3B**).

Subdivision of CC patients belonging to the CXCL16(+) category could also be achieved by LGR6 mRNA analysis (**Figure 3D**). The LGR6(+) category (*n* = 17) showed a 3.9-fold higher recurrence risk compared to the LGR6(-) category (*n* = 31) when followed for 5 years and 3.7 when followed for 12 years (*p*=0.004 at both timepoints; **Table 2**). Corresponding figures for decrease in mean survival time was 18 months at 5 years and 48 months at 12 years after surgery (*p*=0.002 at both timepoints).

Finally, we used LGR6 mRNA analysis to further divide a patient group expressing high or intermediate levels of CEA mRNA in their LNs as well as high levels of CXCL16 (CEA(+)/CEA(int)/CXCL16(+) group). Patients in the LGR6(+) category (*n* = 14) showed a 3.5-fold higher recurrence risk compared to the LGR6(-) category (*n* = 22) when followed for 5 years and 3.8-fold at a follow-up time of 12 years (*p*=0.02 and *p*=0.01, respectively). The Kaplan-Meier survival analysis was associated with a decrease in mean survival time of 17 months in 5 years and 57 months in 12 years after surgery (*p*=0.01 and *p*=0.008, respectively; **Figure 3F**).

### 3.5 LGR6 mRNA expression levels of lymph nodes of colon cancer patients in relation to TNM stage

**Figure 4:**
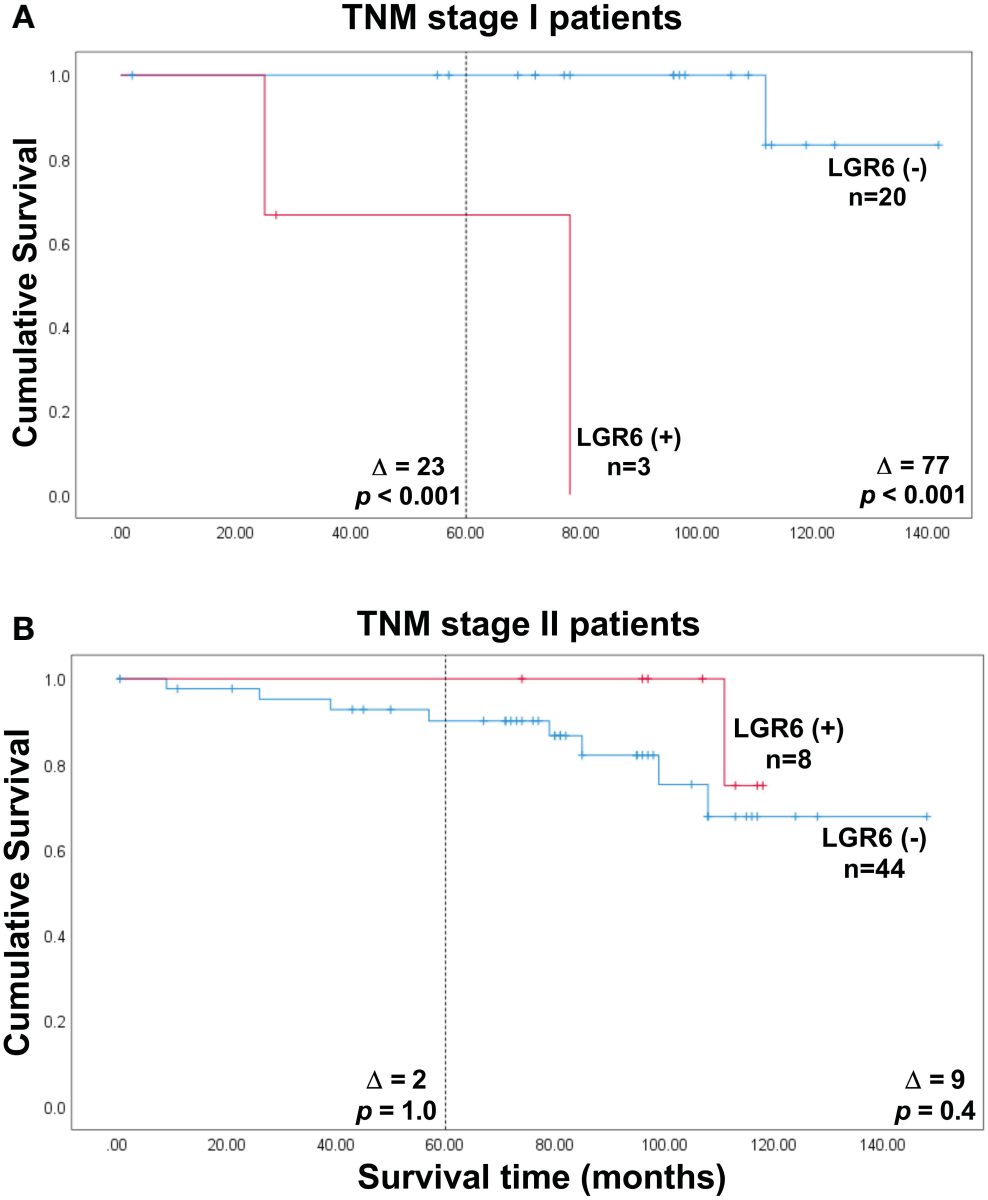
Cumulative survival curves according to Kaplan-Meier of colon cancer patients belonging to either of the two categories LGR6(+) and LGR6(-) defined as patients with a LGR6 mRNA level in the highest lymph node above respectively below the cut-off 0.0471 LGR6 mRNA copies/18S rRNA unit. **(A)** the 23 TNM stage I colon cancer patients. **(B)** the 52 TNM stage II patients. The numbers next to the curves indicate the number of patients in the category. The difference between mean survival time without recurrence between the categories are given as Δ-values. The *p*-values are from log rank test analysis of survival data. The dashed line indicates 5 years of observation after surgery.

**Table 3** shows how LGR6(+) and LGR6(-) patients are distributed in relation to different TNM stages and **Figures 4A, B** show Kaplan Meier analysis of TNM stage I and II patients, respectively. As can be seen, there are 3 stage I patients which are LGR6(+) and 20 which are LGR6(-). Two of the LGR6(+) patients have died from their cancer and the third patient from other causes. After 110 months no LGR6(-) patient had died from cancer (**Figure 4A**; Δ = 23 months at 5 years, *p*<0.001). It can safely be concluded that two of these patients are missed by histopathology and also missed by CEA mRNA analysis since they were found to belong to the CEA(-) group (**Table 3**). Thus, LGR6 analysis adds to histopathology and CEA mRNA analysis. There are 8 stage II patients that fall into the LGR6(+) category and 44 patients in the LGR6(-) category of which 4 were found to belong to the CEA(int) and 4 to the CEA(-) groups (**Table 3**). However, no significant difference in recurrence risk or mean survival time was observed between the LGR6(+) and LGR6(-) categories when analysis was confined to LNs of TNM stage II (**Figure 4B**).

**Table 3:** Number of LGR6(+) and LGR6(-) colon cancer patients in different TNM stages divided into different patients groups.

Patient Group	Category^a^	Number of LGR6(+) patients			
		Stage I	Stage II	Stage III	Stage IV
All CC Patients n=121	LGR6(-)	20	44	25	2
	LGR6(+)	3	8	12	7
CEA(+) patients^b^ n=22	LGR6(-)	2	1	5	1
	LGR6(+)	0	0	6	7
CEA(int) patients^c^ n=54	LGR6(-)	10	25	7	1
	LGR6(+)	1	4	6	0
CEA(-) patients^d^ n=45	LGR6(-)	8	18	13	0
	LGR6(+)	2	4	0	0
CXCL16(+) patients^e^ n=48	LGR6(-)	8	13	9	1
	LGR6(+)	2	2	7	6
CEA(int)/CEA(+)/ CXCL16(+) patients^f^ n=36	LGR6(-)	5	9	7	1
	LGR6(+)	0	1	7	6

a CC patients were divided into categories according to LGR6 mRNA level. LGR6(-): the highest lymph node had <0.0471 mRNA copies/18S rRNA unit; LGR6(+): the highest lymph node had ≥0.0471 mRNA copies/18S rRNA unit.

b CC patients with CEA mRNA levels above 3.67 mRNA copies /18S rRNA unit.

c CC patients with CEA mRNA levels between 0.013 and 3.67 mRNA copies /18S rRNA unit.

d CC patients with CEA mRNA levels below 0.013 mRNA copies /18S rRNA unit.

e CC patients with CXCL16 mRNA levels above 7.2 mRNA copies /18S rRNA unit.

f CC patients with CEA mRNA levels above 0.013 mRNA copies /18S rRNA unit and CXCL16 mRNA levels above 7.2 mRNA copies /18S rRNA unit.

### 3.6 No correlation between recurrence risk or survival after surgery and levels of LGR6 mRNA in primary tumors of colon cancer

When CC patients were divided to LGR6(+) and LGR6(−) categories based on the median mRNA level of the primary CC tumors (0.7 mRNA copies/18S rRNA unit) or the 75^th^ percentile as cutoffs, no differences were found between the groups in either the survival time or recurrence risk.

## 4 Discussion

The most important finding of this study is that LGR6 can be used as a complementary biomarker to CEA and CXCL16 to detect CC patients that relapse after surgery who are missed by these markers and by histopathology. LGR6 is useful as a complementary biomarker in mRNA analysis of LNs of patients with CC but not for analysis of the primary tumor. LGR6 mRNA analysis has prognostic value in two different situations 1) if the CC patient has LNs expressing high levels of CEA mRNA and 2) if the CC patient has LNs that do not express CEA mRNA (that is CEA mRNA levels below the cut of level for LNs of control patients). In the former situation LGR6 mRNA levels discriminate between patients with very bad prognosis and those with less bad prognosis. In the latter situation high levels of LGR6 mRNA reveal those relatively few patients that relapse but only express minimal levels or no CEA at all, the CEA(-) group. Of particular interest is that these patients also are missed by histopathology i.e. they belong to TNM stage I and II. In this study, LGR6(+) stage I and II patients constituted 11 patients which is equal to 9% of all patients. LGR6 could detect CC patients at risk in stage I but not in stage II, indicating that the size of the primary tumor does not necessarily reflect the aggressivity of the cancer. Probably genetic features of the tumor have a greater impact. Why does LGR6 detect patients with bad prognosis who are not detected by CEA or CXCL16? We hypothesize that this is due to that LGR6 detect colonic epithelial stem cells which are poorly detected by CEA and CXCL16. Such stem cells also occur to a variable degree in LNs of CC patients. However, LGR6 is not a stem cell specific marker in humans as revealed by studies with monoclonal antibodies (31). LGR6 is also expressed in tumor cells that are more mature, the difference being that CSC express higher levels than more mature cancer cells. Moreover, our cell line studies indicate that LGR6 mRNA is highly expressed in colonic CSC. The CC cell line HCT8 expressed very high levels of LGR6 mRNA, which is in line with the findings by Yan et al., 2016 who found that CSC could easily be isolated from this cell line (32). Another important observation is that LGR6 detects a subpopulation of tumor cells that is not detected by LGR5 since LGR6 identified CC patients at high risk with low CEA levels that were not identified by LGR5 (10). Despite the similarity between the structure of LGR6 and LGR5 (33, 34), LGR6 was barely detected in fibroblasts, in contrast to LGR5, which was expressed at high levels suggesting differences in function between the two CSC markers. In this study we have used PCR primers and a probe that detect all three splice forms of LGR6 but do not cross-react with mRNA for LGR5 or LGR4. An interesting possibility, that has not been explored, is that any of the three splice forms could have a different specificity pattern.

LGR6 mRNA levels correlate with CEA and CXCL16 mRNA levels in stage III and IV patients and LGR6 correlates significantly, but less strongly, with LGR5 and LGR4 and in nearly all TNM stages. The relationship between the three LGRs is complex and is not fully understood. LGR4 and LGR6 show a closer expression pattern than LGR5 and LGR6 as shown in this study and our previous study (10). It was noted that LGR6 protein can bind to LGR4 protein and LGR5 protein possibly indicating that LGRs form complexes with each other (19) that can positively or negatively regulate the Wnt/β-catenin pathway. Complex formation between LGRs may be responsible for the contradictory results seen by different groups using LGRs as prognostic marker in cancer including CC.

LGR6 promotes CRC cell proliferation and migration *in vitro* by activating the PI3K/AKT signaling pathway and was suggested to serve as a predictive biomarker of CRC primary tumors for bad prognosis and a therapeutic target for patients with advanced stages of CRC (20). Moreover, LGR6 is implicated in the growth and proliferation of several cancer types, including gastric and colon cancer and is also attributed with cancer therapy resistance (20, 35–38).

It is unlikely that the results presented here would have been possible to obtain by histopathology or even immunohistochemistry with specific antibodies supported by artificial intelligence (AI) deep learning algorithms, because only a small portion of the LN volume is analyzed by these methods (39, 40). We showed in a previous study that disseminated tumor cells are heterogeneously distributed in the LN and metastases can be missed if only a small volume is analyzed (41). In the present study the molecular technique qRT-PCR was used to analyze extracts from as much as half the LN thereby strongly increasing the probability of detecting LGR6 mRNA from cancer stem cells. Another complicating factor for the microscopic methods is selection of LNs for examination of presence of stem cells and assessment of stem cell numbers. The LNs of a single patient can differ considerably regarding the number of tumor cells and the risk factors these cells express (6). The fact that current guidelines for determination of metastasis status (pN-stage) requires examination of a minimum of 12 LNs points out the fact that LNs of one patient vary considerably in tumor burden (42, 43). Determination of LGR6 mRNA levels is readily done in several LNs in a fast and objective manner.

The novel results of this study, obtained with our well-studied clinical material of CC patients, need to be validated in a larger clinical material, which should also include patients with rectal cancer. Moreover, preferably all LNs collected from a patient should be included in the study.

## 5 Conclusion

We conclude that LGR6 mRNA analysis of LNs from CC patients can serve as an important complement to CEA- or CXCL16 mRNA analysis detecting cancer stem cells which express very low levels or no mRNA for these two markers. Moreover, it appears to be difficult to identify cancer cells in these LNs by histopathology either because the number of cancer cell is very low or that the CSCs are very unevenly distributed in the LN tissue. LGR6 has a different expression pattern than the CSC marker LRG5 and could detect other patients at risk. Using LGR6 mRNA analysis will help to identify additional patients which would benefit from adjunct therapy.

## Data availability statement

The original contributions presented in the study are included in the article. Further inquiries can be directed to the corresponding author.

## Ethics statement

The study was approved by the Local Ethics Research Committee of the Medical Faculty, Umeå University, Umeå, Sweden (registration number: 03-503; date of approval: 3 December 2003 and registration number: 2023-01396-01; date of approval; 3rd of Mai 2023). The studies were conducted in accordance with the local legislation and institutional requirements. The participants provided their written informed consent to participate in this study.

## Author contributions

HE: Writing – original draft, Data curation. MA: Writing – review & editing, Data curation. HI: Writing – review & editing, Data curation. FZ: Writing – review & editing, Supervision. AG: Writing – review & editing, Supervision. LO: Writing – review & editing, Data curation. GL: Writing – review & editing, Data curation. MH: Writing – review & editing, Writing – original draft, Resources, Data curation, Conceptualization. SH: Writing – review & editing, Writing – original draft, Visualization, Data curation, Conceptualization. BS: Writing – review & editing, Writing – original draft, Visualization, Supervision, Resources, Project administration, Data curation, Conceptualization.
